# Factors associated with length of stay and treatment outcome of Ebola patients treated at an Ebola treatment center in Sierra Leone during the peak period of the West African Ebola outbreak 2013–2016

**DOI:** 10.1186/s13690-021-00653-w

**Published:** 2021-07-12

**Authors:** Jia Bainga Kangbai, Christian Heumann, Michael Hoelscher, Foday Sahr, Guenter Froeschl

**Affiliations:** 1 Center for International Health, University of Munich (LMU), Munich, Germany; 2 Department of Environmental Health Sciences, Njala University, Freetown, Sierra Leone; 3 School of Public Health and Tropical Medicine, Tulane University, New Orleans, USA; 4 Department of Statistics, University of Munich (LMU), Munich, Germany; 5 Division of Infectious Diseases and Tropical Medicine, University Hospital, LMU, Munich, Germany; 6 Department of Microbiology, College of Medicine and Allied Health Sciences, University of Sierra Leone, Freetown, Sierra Leone; 7 The 34 Military Hospital, Wilberforce, Freetown, Sierra Leone

**Keywords:** Case fatality rate, Clinical factors, Ebola virus disease, Epidemic, Length of stay, Outbreak, Sociodemographic, Symptomatic period, Treatment outcome, Viral Haemorrhagic fever

## Abstract

**Background:**

The World Health Organization (WHO) declared the West Africa Ebola epidemic as a Public Health Emergency of International Concern in August 2014. During the outbreak period, there were calls for the affected countries to construct Ebola treatment centres and reliable diagnostic laboratories closer to areas of transmission in order to improve the quality care of Ebola Virus Disease (EVD) patients. Delay in seeking treatment has been reported to have led to poor treatment outcome of EVD patients. Sierra Leone recorded more than 8000 probable and confirmed cases and more than 4000 EVD -related deaths nation-wide.

**Methods:**

In this retrospective study, we investigated the effects of treatment delay, length of symptomatic period, EVD patients’ sex, age, occupation, region of residence, and clinical characteristics on the treatment outcome of 205 laboratory-confirmed EVD patients who were admitted at the Kenema Government Hospital Ebola Treatment Center (KGHETC) from 13/09/2014–26/11/2014; i.e. during the peak of 2013–2016 EVD outbreak in Sierra Leone. Specifically also, we determined the factors that were associated with the length of stay for EVD treatment for patients who were discharged alive.

**Results:**

Majority (66.3%, *n* = 205/309) of the 309 suspected EVD patients with medical records at the KGHETC triage during the period under review were tested positive for EVD using reverse-transcriptase-polymerase chain reaction (RT-PCR) and had a definitive treatment outcome. Few (33.7%, *n* = 104/309) suspected EVD patients were not included in our analysis and were classified thus: 29.1% (*n* = 90/309) suspect EVD cases with negative RT-PCR results, 4.5% (*n* = 14/309) suspect cases with non-available RT-PCR result.

Of the 205 patients, 99 (48.3%) had a fatal outcome. For EVD patients that survived, we recorded a significant association (− 0.06, 95% Confidence Interval (CI) = − 0.14 – - 0.02, *p* = 0.004) between the Length of Stay (LOS) and for each kilometer travelled to seek treatment at the KGHETC. However, the association between EVD patients that were low skilled workers (− 5.91, 95% CI = − 24.60 – 12.79, *p* = 0.73), EVD patients who were children and pupils in junior school (− 0.86, 95% CI = − 12.86 – 11.14, p = 0.73), health seeking delay for EVD patients who resided in Kenema District where the KGHETC was located (− 0.49, 95% CI = − 0.12 – 1.09, *p* = 0.24), sex (− 1.77, 95% CI = − 8.75 – 5.21, *p* = 0.50), age (0.21, 95% CI = − 0.36 – 0.77, *p* = 0.57), referral status (1.21, 95% CI = − 17.67 – 20.09, *p* = 0.89) and the LOS in surviving patients were not statistically significant.

**Conclusion:**

The high LOS for either treatment outcome for EVD patients that resided in the district in which the EVD treatment facility was located compared to those patients from other districts implies that health authorities should consider intensive health education with high priority given to seeking early EVD treatment, and the construction of strategic ETCs as important components in their response strategy.

## Background

The World Health Organization (WHO) declared the West Africa Ebola epidemic as a Public Health Emergency of International Concern in August 2014 and requested that countries with high Ebola Virus Disease (EVD) transmission to make available treatment centres and reliable diagnostic laboratories close to the areas of EVD transmission [1, 2]. Sierra Leone was one of the countries that was greatly affected by the 2013–2016 EVD outbreak; recording more than 8000 probable and confirmed cases and more than 4000 EVD-related deaths nation-wide [3]. Different Case Fatality Rate (CFR) values were reported for different locations and Ebola Treatment Centers (ETCs) during the 2013–2016 EVD outbreak [4–8]. Many facility-based studies have reported that age, higher viremia, and clinical symptoms such as diarrhea, weakness, conjunctivitis and confusion are associated with high CFRs [9–13]. However, the role of health seeking delays, which is also related to the distance to an ETC, the geographical region, treatment delay, referral pathway of EVD patients, alongside with the sociodemographic and clinical characteristics of EVD patients on EVD treatment outcomes still remain unclear. An online search in the PubMed library on the search terms [Ebola] and [delay] reveals merely 57 hits (as per 08 May 2020), of which none dealt conceptually with health seeking delays. In Sierra Leone, ETCs provided 60% [14] of the necessary treatment beds during the West Africa EVD outbreak in 2013–2016. There has been speculation that the potential spread of Ebola Virus (EV) prior to the admission of EVD patient for treatment during the West African EVD outbreak may be attributed to the low number of treatment beds that were available at the ETCs [ 5, 15–17]. According to Fitzpatrick G and colleagues, EVD patients who traveled 0 to 40 km to seek Ebola treatment at the Kailahun case management center recorded higher CFR (60%) compared to EVD patients who traveled > 200 km (CFR = 40%) [4]. There is also a paucity of information on determinants for the LOS in ETCs during EVD treatment. In this study, we investigated the effect that health seeking delays, sociodemographic and clinical characteristics of EVD patients had on EVD treatment outcomes in the specific context of the Kenema District in Sierra Leone. Furthermore, we believe that an understanding of these factors will corroborate community measures in comparable future situations that aim at improving treatment-seeking behavior among EVD patients, provide an understanding of the most appropriate and accepted locations for ETC construction as well as offer the best ETC design that will guarantee safe treatment and care for EVD patients. We are of the opinion that an understanding of the role played by health seeking delay for those patients who were treated at the KGHETC, alongside that of the sociodemographic and clinical characteristics of EVD patients on the treatment outcome can also serve as an incentive to seek early treatment and hence obtain a better treatment result. Also, considering the fact that EVD outbreaks usually occur in resource-constrained settings in Africa, we believe that it is important to know the link between these factors and the CFR since EVD patients in these settings often have to compete for the available bed space and clinical attention following their diagnosis. Generally, comparing CFRs across different subpopulations of EVD patients, regions, and ETCs on the basis of EVD patient’s sex, age, occupation, region of residence and clinical characteristics alone is not sufficient. If CFRs are to be understood and compared across different subpopulations of EVD patients, regions, and ETCs, such comparison should take into account the LOS, health seeking delay, length of symptomatic period, patient referral pathway, and distance to ETC alongside the various factors that influence them. The limited access to local EVD health care was perceived during the 2013–2016 EVD outbreak to have an impact on treatment seeking and referral [18]. In Africa, where most EVD outbreaks occur, many people who become infected usually have to travel long distances on bad road networks and in overcrowded ambulances to seek EVD treatment. Those infected people who stayed at home because of these challenges eventually contributed to the community transmission of EVD due to their long delay in seeking treatment, while those that survive the journey frequently die upon arrival at the ETC due to their poor health status [5].

In this retrospective study, we investigated the effect health seeking delay by EVD patients residing in Kenema District were the KGHETC was located, length of symptomatic period, EVD patients’ sex, age, occupation, region of residence, and clinical characteristics had on the treatment outcome of 205 laboratory-confirmed EVD patients at the KGHETC between the 2013–2016 EVD outbreak.

## Methods

### Study design

In total, 309 suspected EVD patients presented themselves consecutively at the KGHETC during the study period covering 13th September 2014 to 26th November 2014; this was the peak of the 2013–2016 EVD outbreak in Sierra Leone. We decided to analyse the Ebola treatment outcome data for the period under review because we believed that the incidence and case fatality rates for diseases vary during an outbreak period. Incidence and case fatality rates for diseases are general high during the onset of an outbreak due to lack or improper preparedness but such rates start to decline gradually as the outbreak progresses due to the availability of both the preventive and treatment options for the disease in question. We analysed the Ebola treatment outcome data for the KGHETC during the period under investigation in order to avoid both lead-time and length-time biases which may have being brought about as a result of the inclusion of EVD cases from different time periods during the outbreak. We believed that to prevent length-time bias in this study, we should analysed all Ebola treatment outcome data that were recorded during a specific period (in our case during the peak period of the outbreak) of the outbreak rather during the entire outbreak period irrespective of the condition in which these outcomes occurred. We believed that by selecting a specific period in the outbreak the outcomes that occurred during the targeted period were influenced by the same condition and hence may have prevented the accumulation of excess asymptomatic EVD cases (length-time bias) which can give rise to an overestimation of survival duration due to early detection (lead-time bias) for one set of EVD cases over the others.

Lead-time bias produce an overestimated survival duration of a disease due to the early detection by screening rather than by clinical diagnosis; while length-time bias produce an overestimated survival duration of a disease due to the relative excess of slowly progressing cases.

We analysed the complete medical records containing the sociodemographic and clinical characteristics of 205 confirmed EVD patients out of the 309 suspect cases, who were then admitted for treatment at the KGHETC. We described distance to the KGHETC, EVD patient referral status, length of symptomatic period, delay in seeking treatment, and EVD patients’ sex, age, occupation, region of residence, and clinical characteristics, and the correlations between these factors and the facility-based EVD treatment outcome. An EVD confirmed patient in this study was a person fulfilling case definition criteria and whose full blood, serum, or plasma specimen had tested positive for EVD by RT-PCR assay. Sociodemographic and health seeking delay data are presented in a descriptive fashion for both confirmed cases and non-cases. Patients in whom RT-PCR for Ebola virus was not conducted, or revealed a negative or indeterminate result and patients in whom records on the treatment outcome are missing were excluded. Only laboratory-confirmed EVD patients with complete treatment outcomes (cured or died) were further analysed in this study.

### Study setting

**Fig. 1:**
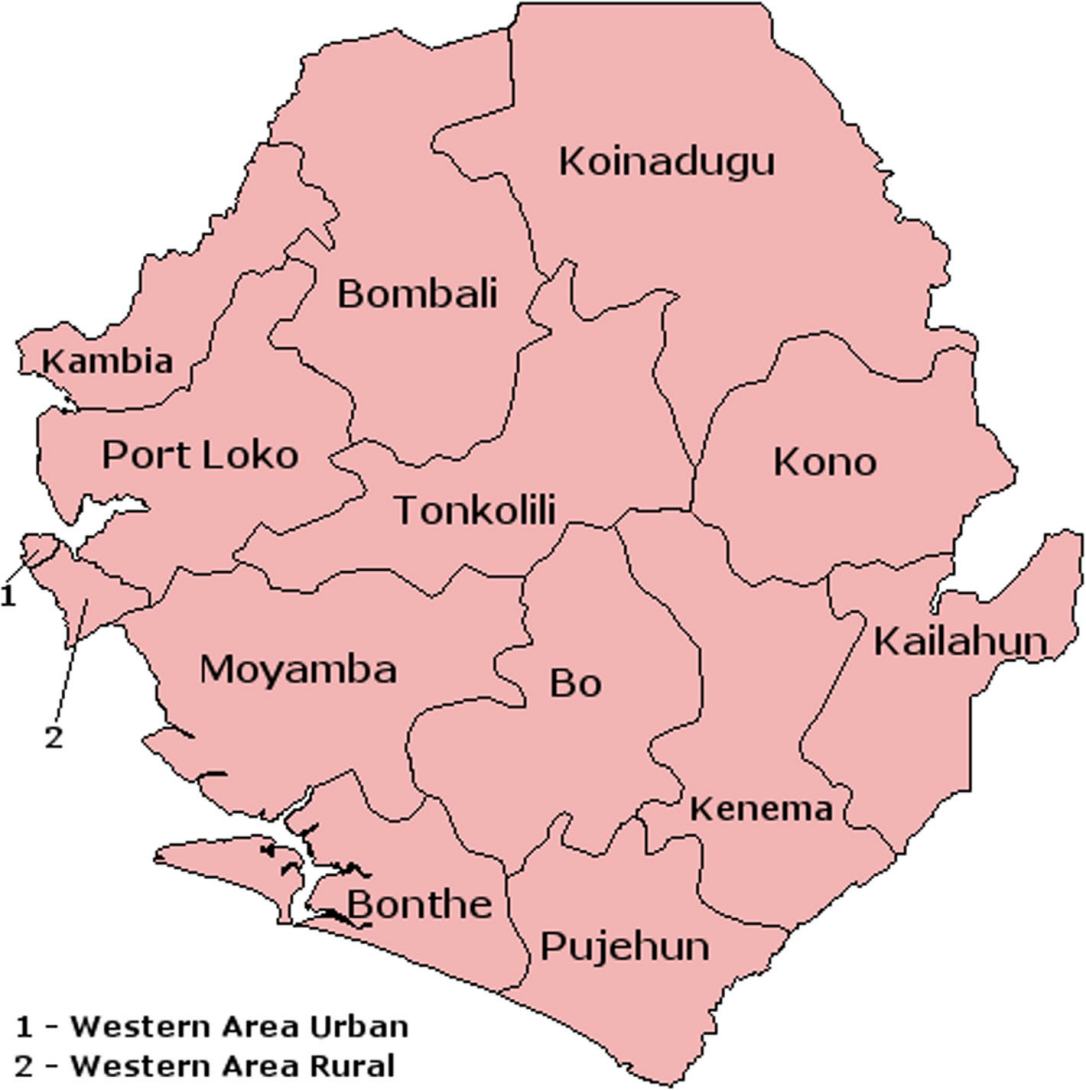
District map of Sierra Leone

The Kenema Government Hospital (KGH) where the KGHETC was located is the largest government district hospital in eastern Sierra Leone (Fig. 1).

The map of Sierra Leone shows the various districts including those where patients treated at KGHETC were residing during the 2013–2016 EVD outbreak. Source: Wikipedia (https://en.wikipedia.org/wiki/Districts_of_Sierra_Leone).

Prior to the 2013–2016 EVD outbreak, it served as the national referral center for Lassa fever [19], had 472 staff and volunteers; and was equipped with a surgical, adult medicine, pediatric, and maternity wards [20]. During the 2013–2016 EVD outbreak, KGH had 350 beds and catered for 670,000 patients [21]. During the early period of its operation, the KGHETC only treated EVD patients from Kenema and Kailahun districts. As the outbreak progressed, KGHETC services were later extended to EVD cases from other regions of the country. All Kenema District EVD patients were diagnosed for EVD at the KGHETC triage, whereas non Kenema district EVD patients came from outside Kenema District and were diagnosed and subsequently admitted at the KGHETC. The EVD patients whose medical records we analysed in this study either self-reported at the KGHETC triage as suspected EVD case or were brought to the KGHETC triage by personnel attached with the National Ebola Response Center (NERC).

### Treatment protocol

All EVD patients in this study whose medical records were analysed were routinely provided oral rehydration salts (ORS) and other electrolyte replacement supplements upon commencement of treatment. Based on their presenting symptoms, the EVD patients were also provided with acetaminophen or ibuprofen, ciprofloxacin or cefixime, and naphthoquine phosphate for pain, bacterial, and malaria infections respectively. Ranitidine or omeprazole were also given to EVD patients who experienced abdominal pain. The EVD treatment protocols that were practiced by KGHETC clinicians did not change during the course of this study, and they were performed in accordance with the WHO protocol of urgent interim guidance for EVD case management for viral haemorrhagic fever [22].

### Ethics review

We obtained approval for this study from the Sierra Leone Ethics and Scientific Review Committee (Opinion Date March 29, 2017) and the Institutional Review Board at the Ludwig-Maximilians-Universität in Munich, Germany (Opinion No. LMU 17–582). We were granted ethical clearance and waived the requirement to obtain individual informed consent from the EVD patients on the basis that we were analyzing data that were to be presented in an aggregated form.

### Data collection and processing

Our dataset was curated by trained clinicians, Ebola surveillance officers, and data clerks that were attached to the KGHETC. Initially, the medical records of the EVD patients were recorded on hard copies of Case Report Forms (CRF). The data on the CRF were later transferred to a Microsoft Excel file [23] for data cleaning and statistical analysis. We anonymized the medical records of all EVD patients that were analysed in this study and later stored the data in a computer that can only be accessed with a secured password. The WHO case definition for EVD was used by the KGHETC to consider EVD suspects for testing [24].

### Variables and statistical analysis

R software package version 3.3.1 [25] was used for all descriptive and regression analyses in this study. The *p*-values < 0.05 were considered significant for all two-sided statistical tests. We present our descriptive analysis either as frequencies, proportions, means and standard deviations (for normally distributed continuous variables), or medians and interquartile ranges (IQR) for non-normally distributed continuous variables. Chi-square tests (when our sample sizes are 5 and above) and Fisher’s exact test (when our sample sizes were less than 5) were used to determine correlations between categorical variables. Delays and time periods were calculated by subtracting different variables of time point data from each other.

**Fig. 2:**
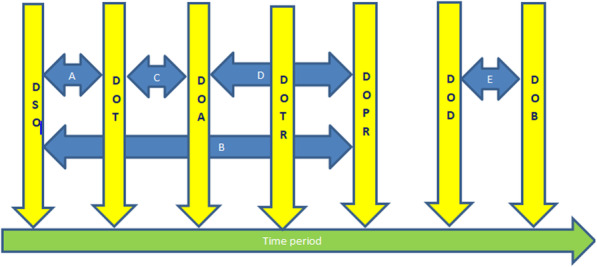
Treatment associated time points and periods for Ebola Virus Disease patients treated at the Kenema Government Hospital Ebola Treatment Center during the peak period of the West Africa Ebola outbreak 2013–2016

The descriptive part comprises socio-demographic variables (sex, age, occupation, referral status, distance travel and location of residence, potential source of EVD infection), clinical patient characteristics (blind, mutism, anorexic, and admitted in a bad state) and various time periods (Table 1 and Fig. 2).

**Table 1:** Full text meaning of abbreviations, description of time periods for Ebola Virus Disease patients treated at the Kenema Government Hospital Ebola Treatment Center and their respective placement in Fig. 2

Abbreviation and meaning	Time period/Description	Fig. 2
CHD: Cadaver Handling Delay	This was the period between the date an EVD patient died while undergoing treatment to the date of burial	Arrow E
DOA	Date of admission of EVD patient	
DOB	Date of burial of EVD patient	
DDP: Delay in the Discharge of Patient	This was the period between the date an EVD patient was cured to the date he/she was discharged	
DOD	Date of death of EVD patient	
DOT	This was the date when the first EVD laboratory test was conducted	
DOPR	This was the date when an EVD patient that was successfully treated was discharged	
DOTR	Date of release of EVD test result	
DSO	This was the date when EVD symptom/s were first observed in the patient	
DTI: Discharge Testing Interval	This was the time period between the date an EVD patient was cured to the date his/her final laboratory test results were released	
HSD: Health seeking delay for all EVD patients irrespective of district of origin	This was the period between the date an EVD patient showed symptoms of EVD to the date he/she was admitted at the KGHETC for treatment irrespective of the district of origin of the EVD patient	Arrow A
HSDKD: Health Seeking Delay for patient residing in Kenema District	This was the period between the date an EVD patient who was residing in Kenema District started showing symptoms of EVD to the date he/she sought treatment at the KGHETC	
HSDNK: Health Seeking Delay for patient residing outside Kenema District	This was the period between the date an EVD patient who was not residing in Kenema District started showing symptoms of EVD to the date he/she sought treatment at the KGHETC	
LOS: Length of stay	This was the period between the date of admission and date of discharge of the EVD patient	Arrow D
LOSA: Length of Stay for EVD Patient who Survived Treatment	This was the period between an EVD patient started developing EVD symptoms to the date it took for he/she to be successfully treated and discharged from the KGHETC	Arrow B
LSP: Length of Symptomatic Period	This was the period between the date of EVD symptoms onset to the time the date of the first follow-up laboratory test result was obtained prior to the discharge of the patient, as a proxy time point indicating resolution of symptoms	
RRD: Return of Result Delay	This was the period between the date of the initial EVD laboratory tests were done to the date of the EVD laboratory test results became available and the patient subsequently admitted	Arrow C

We used logistic regression analysis to determine the relationship between in-facility mortality and EVD health seeking delay for those EVD patients who resided in Kenema District, sex, age, occupation, and the distance travelled to the ETC. We did not include health seeking delay for EVD patients who were residing outside Kenema District but were treated at the KGHETC because this subpopulation of patients had already started seeking treatment prior to their arrival at the KGHETC. We categorised occupation into the following levels; children and pupils in junior school), lower skilled workers (farmers, carpenters, mechanics, drivers, and EVD patients with unspecified jobs), and higher skilled workers (students in higher tertiary schools and institutions, medical personnel and teachers).

We also performed two linear regression analyses to determine the association between the average LOS (separately for EVD patients who were discharged alive, and for EVD patients who died during EVD treatment) and EVD treatment delay, sex, age, occupation, referral status, and the distance to ETC.

## Results

### Study patients

**Fig. 3:**
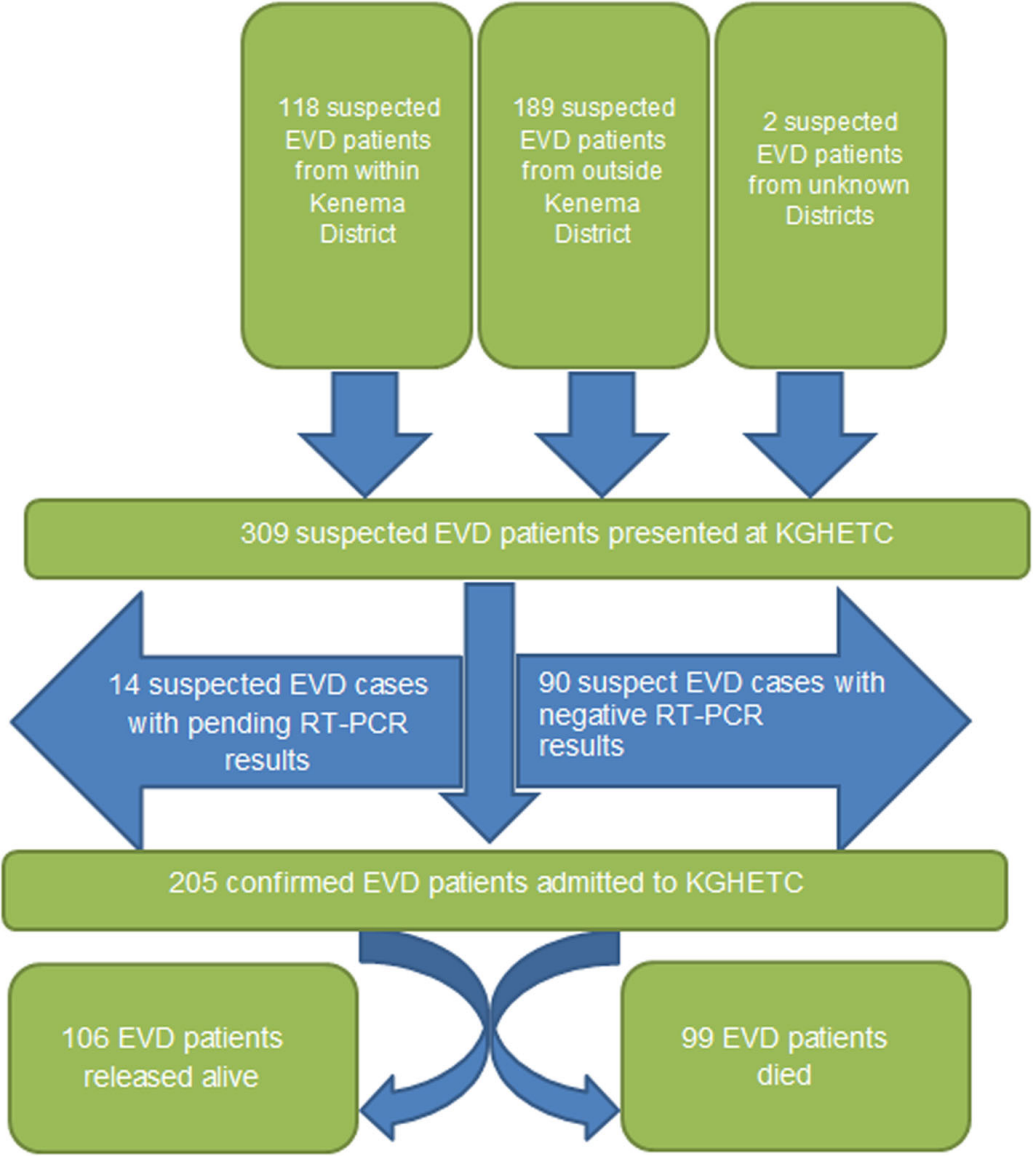
Intake and treatment outcome for Ebola Virus Disease patients who were treated at the Kenema Government Hospital Ebola Treatment Center during the peak period of the West Africa Ebola outbreak 2013–2016

Out of 309 suspected EVD patients with medical records at the KGHETC triage during the period under review, a majority (66.3%, *n* = 205/309) were tested positive for EVD using RT-PCR and had a definitive treatment outcome. The remaining 104 (33.7%) suspected EVD patients that were not included in our analysis were classified as: 29.1% (*n* = 90/309) were suspect EVD cases with negative RT-PCR results, in 4.5% (*n* = 14/309) of the suspect cases the RT-PCR result was not available. Patients with negative test results were either released after spending few days under observation at the KGHETC or were transferred to the regular hospital wards for other treatment (Fig. 3).

The flowchart represents the intake of patients and the outcome of patient trajectory of EVD cases who visited the KGHETC triage from September 2014 to November 2014.

A slight majority (53.2%, *n* = 109/205) of the confirmed EVD patients were female. The most prominent age group was 15 years to below 25 years (23.9%, *n* = 49/205). The median age of the EVD patients was 25 years (Interquartile range = 16 years – 35 years). Of those EVD patients with occupational records, majority (60.3%, *n* = 123/204) were low skilled workers including traders, carpenters, farmers, drivers or individuals with unspecified jobs.

The median distance travelled to seek EVD treatment was 193.0 km (IQR = 1.0 km − 307.0 km) and the majority (73.7%, *n* = 151/205) of the EVD patients resided outside Kenema District where the KGHETC was located; Bombali District (8.8%, *n* = 18/205), Tonkolili District (3.4%, *n* = 7/205), Moyamba District (7.3%, n = 15/205), Kono District (1.0%, *n* = 2/205), Western Urban (33.2%, *n* = 68/205), Western Rural (5.9%, *n* = 12/205), Port Loko District (10.2%, *n* = 21/205), and Pujehun District (3.9%, *n* = 8/205). Only 26.3% (*n* = 54/205) of the EVD cases resided in Kenema District and were admitted directly after going through triage procedures at the KGHETC.

The following are the median, for health seeking delay by EVD patients irrespective of district of referral: 6.0 days (IQR = 3.00 days - 11.00 days), return of result delay: 3.00 days (IQR = 2.00 days - 4.00 days), discharge testing interval 0.00 day (IQR = 0.00–2.00 days), delay in the discharge of EVD patients: 1.00 day (IQR = 1.00 day – 3.00 days), cadaver handling delay: 0.00 day (IQR = 0.00 day – 1.32 days), and length of EVD symptomatic period: 14.0 days (IQR = 9.00 days - 20.00 days) (see also Table 2).

**Table 2:** Treatment associated time periods for Ebola Virus Disease patients who were treated at the Kenema Government Hospital Ebola Treatment Center during the peak period of the West Africa Ebola outbreak 2013–2016

Time periods	Minimum (days)	25% Percentile (days)	Median (days)	75% Percentile (days)	Maximum (days)	Number of missing data
Length of Symptomatic Period	2.00	9.00	14.00	20.00	23.00	21
Health seeking delay for all EVD patients	0.00	3.00	6.00	11.00	24.00	82
Health Seeking Delay for Kenema patients	0.00	2.00	8.00	11.00	18.00	18
Health Seeking Delay for non Kenema patient	0.00	3.00	6.00	11.00	24.00	24
Return of Result Delay	0.00	2.00	3.00	4.00	8.00	12
Discharge Testing Interval	0.00	0.00	0.00	2.00	8.00	19
Delay in the Discharge of Patient	0.00	1.00	1.00	3.00	9.00	5
Cadaver Handling Delay	0.00	0.00	0.00	1.32	2.87	11
Overall Length of stay for EVD treatment	1.00	3.00	6.00	11.00	24.00	18
Length of Stay for EVD Patient who Survived Treatment	1.00	4.00	10.50	14.00	24.00	9

**Fig. 4:**
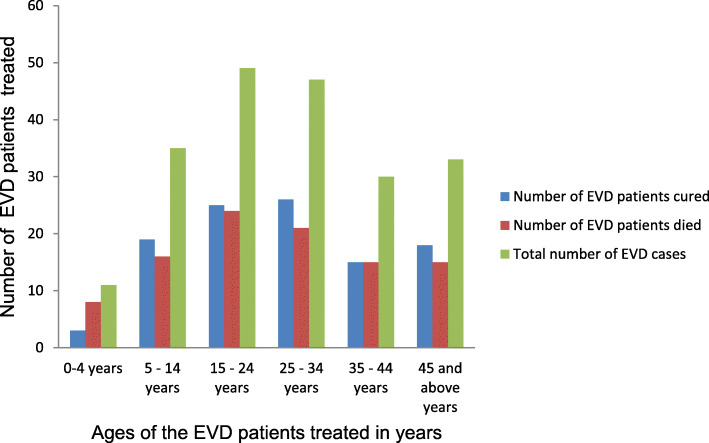
Age distribution Ebola Virus Disease patients who were treated at the Kenema Government Hospital Ebola Treatment Center during the peak period of the West Africa Ebola outbreak 2013–2016

The overall CFR at the KGHETC was 48.3% (*n* = 99/205); male EVD patients recorded an insignificantly higher CFR (52.1%, *n* = 50/96, *p* = 0.379) compared to female EVD patients (CFR = 45.0%, *n* = 49/109). With the exception of our regression models in which age is a continuous variable, we categorised age into age groups in all bivariate analysis for better presentation. EVD patients belonging to the age group 0 year to 4 years (CFR = 72.7%, *n* = 8/11, *p* = 0.682) recorded an insignificantly higher CFR compared to EVD patients in the other age groups (Fig. 4).

EVD patients belonging to the age group 15 to 24 years recorded the highest number of EVD patients that died in this study while the age group 0 to 4 years recorded the lowest number of EVD patients that died.

**Fig. 5:**
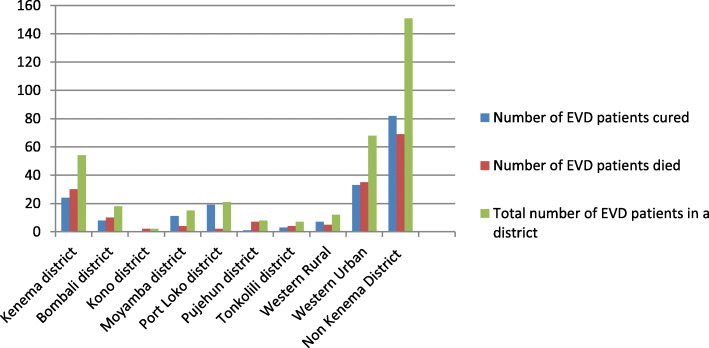
Distribution of treatment outcome Ebola Virus Disease patients who were treated at the Kenema Government Hospital Ebola Treatment Center during the peak period of the West Africa Ebola outbreak 2013–2016 by district of origin

The relationship between the different occupation levels and their respective CFRs was not statistically significant (*p* = 0.534). Of those EVD patients with employment records, low skilled workers recorded the highest CFR (51.2%, *n* = 63/123). Overall, the majority (73.7%, *n* = 151/205, CFR = 45.7%, *n* = 69/151, *p* = 0.278) of the EVD patients came for treatment from outside Kenema District where the KGHETC was located compared to those who were admitted directly from within Kenema District via the KGHETC triage (26.3%, *n* = 54/205, CFR = 55.6%, *n* = 30/54); the CFR for the EVD patients that came from outside Kenema District was lower than those who were admitted directly from within Kenema, but this difference was not significant. There was a statistically significant association between CFRs and the district of residence of EVD patients (*p* = 0.002). The CFRs for Kono (100%, *n* = 2/2), Pujehun (87.5%, *n* = 7/8), Tonkolili (57.1%, *n* = 4/7), Kenema (55.6%, n = 30/54), Bombali (55.6%, *n* = 10/18), Western Urban (51.5%, *n* = 35/68), Western Rural (41.7%, n = 5/12), Moyamba (26.7%, n = 4/15), and Port Loko Districts (9.5%, n = 2/21) (Fig. 5).

Distribution of the treatment outcome for EVD cases treated at the KGHETC for the study period by districts, and whether the EVD patient was referred for treatment from within Kenema District or from outside Kenema District. The number of EVD cases from Western Urban was higher than all the other districts that sent EVD patients for treatment at the KGHETC in this study.

### EVD patients clinical characteristics and potential source of infection

We determined the stratified CFRs for those EVD patients who reported their potential source of infection. There was no significant association between the CFR and the potential source of EVD infection: the CFR for those EVD patients that had direct physical contact with other EVD patients was 28.4% (*n* = 23/81, *p* = 0.444), for having shared apartment with a case 29.7% (*n* = 11/37, *p* = 1.000), for coming in contact with the bodily fluid of a case 32.4% (*n* = 12/37, *p* = 0.816), for having shared clothes with a case 40.0% (n = 2/5, *p* = 0.634), for having attended a funeral 25.0% (n = 1/4 p = 1.000), or having sucked breast milk of an infected mother 75.0% (*n* = 3/4 *p* = 0.073), with a case being either a suspected, probable, or confirmed EVD patient. There was also no significant association between the CFR and whether a given EVD patient was related to either a suspected, probable or confirmed EVD patient already admitted at the KGHETC. The CFR for EVD patients who were related to either a suspected, probable or confirmed EVD patient admitted at the KGHETC was 43.6% (*n* = 17/39) while the CFR for those EVD patients who are not related to either a suspected, probable or confirmed EVD patient admitted at the KGHETC was 50.8% (*n* = 32/63, *p* = 0.543) (Table 3).

**Table 3:** Potential Sources of Ebola Virus Disease infection and Case Fatality Rates for Ebola Virus treatment at Kenema Government Hospital Ebola Treatment Center, Sierra Leone, during the peak period of the West Africa Ebola outbreak 2013–2016

Potential source of EVD patient infection	Total EVD patients (%) 205 (100%)	Survived 106 (51.7%)	Died 99 (48.3%)	Case Fatality Rate (%)	P – value _*_
Breast milk	4 (2.0)	1 (0.9)	3 (3.0)	75.0	0.073
Shared clothes	5 (2.4)	3 (2.8)	2 (2.0)	40.0	0.634
Attended a funeral	4 (2.0)	3 (2.8)	1 (1.0)	25.0	1.000
Related to EVD patient	39 (19.0)	22 (20.8)	17 (17.2)	43.6	0.543
Shared apartment	37 (18.1)	26 (24.5)	11 (11.1)	29.7	1.000
Physical contact with EVD patient	81 (39.5)	58 (54.7)	23 (23.2)	28.4	0.444
Contact with EVD fluids	37 (18.1)	25 (23.6)	12 (12.1)	32.4	0.816
Mutism	2 (1.0)	0 (0)	2 (2.0)	100	0.496
Anorexia	1 (0.5)	0 (0)	1 (1.0)	100	0.488
Blindness	1 (0.5)	0 (0)	1 (1.0)	100	1.000

_*:_ *p* - value was obtained by applying Fishers test by comparing the case fatality rates, clinical characteristics and potential source of EVD contact

### Factors associated with dying during EVD treatment

We used logistic regression model to determine the factors that are associated with dying during treatment for 205 EVD patients treated at the KGHETC. We did not determine the effect delay in seeking EVD treatment would have on the odds of dying for EVD patients residing outside Kenema district because some of these patients had begun EVD treatment en route to their admission at the KGHETC, which would have been a confounding factor. There was a non-significant association between the occupation levels and the odds of dying during EVD treatment. Holding other covariates in the model constant, the odds of dying during treatment for low skilled worker EVD patients and EVD patients who were children and pupils in junior school over the odds of dying for high skilled worker EVD patient were 1.52 (95% CI = 0.32–8.07, *p* = 0.61) and 0.73 (95% CI = 0.11–4.86, *p* = 0.74) respectively. Additionally, holding other covariates in the model constant, there was a non-significant odds of dying during treatment for male EVD patient over the odds of dying for female EVD patient was 1.72 (95% CI = 0.53–5.84, *p* = 0.37). There was no association between dying during EVD treatment and irrespective of whether or not there was a year increase in the age of an EVD patient undergoing treatment at KGHETC (AOR = 1.00, 95% CI = 0.93–1.07, *p* = 0.98), as well as for each day that passed without an EVD patient residing in Kenema district seeking treatment at KGHETC (AOR = 1.00, 95% CI = 0.90–1.11, *p* = 0.97). There was a non-significant trend for decreased odds of dying for each kilometer travelled to seek treatment at KGHETC (AOR = 0.99, 95% CI = 0.98–1.01, p = 0. 28). Overall, EVD patients who resided within Kenema District had a non-significant decreased odds (AOR = 0.24, 95% CI = 0.02–3.07, *p* = 0.28) of dying during treatment compared to those who resided outside Kenema District (Table 4).

**Table 4:** Determinants of death during Ebola Virus treatment at Kenema Government Hospital Ebola Treatment Center, Sierra Leone, during the peak period of the West Africa Ebola outbreak 2013–2016. Results from a multivariate logistic regression

Patient characteristics	Adjusted OR*	95% CI	
Sex: male vs female (ref)	1.72	0.53–5.84	
Occupation: high skilled (ref) workers			
Low skilled workers	1.52	0.32–8.07	
Children and pupils in junior school	0.73	0.11–4.86	
Delay (in days) of health seeking behavior for EVD patients residing in Kenema District	1.00	0.90–1.11	
Distance (in km) to ETC	0.99	0.98–1.01	
Referral status: residency outside-vs-in (ref) Kenema District	0.24	0.02–3.07	
Age	1.00	0.93–1.07	0.98

*OR* Odds Ratio, *ETC* Ebola Treatment Center, *EVD* Ebola Virus Disease, *CI* Confidence Interval

### Factors associated with LOS for confirmed EVD patients that were discharged alive from KGHETC

We used linear regression model to determine the factors that are associated with the LOS for 105 EVD patients admitted to KGHETC as confirmed EVD cases and that were eventually discharged alive. There was a non-significant association between occupation levels, sex, age, EVD patients who resided in Kenema district, delay in seeking EVD treatment, and the LOS for confirmed EVD patients admitted to KGHETC and eventually discharged alive after treatment. Adjusting for other covariates in the model, EVD patients who were low skilled workers (− 5.91, 95% CI = − 24.60 – 12.79, *p* = 0.73), children and pupils of junior schools (− 0.86, 95% CI = − 12.86 – 11.14, p = 0.73), male EVD patients (− 1.77, 95% CI = − 8.75 – 5.21, *p* = 0.50) have non-significantly reduced LOS for confirmed EVD patients admitted to KGHETC and eventually discharged alive after treatment compared to high skilled EVD patients and female EVD patients respectively. However, there was a significant association between the distance traveled to seek EVD treatment and the LOS for confirmed EVD patients admitted to KGHETC and eventually discharged alive after treatment. Those EVD patients who traveled longer distance to seek EVD treatment (− 0.06, 95% CI = − 0.14 – - 0.02, *p* = 0.004) have significantly reduced LOS before they were discharged alive compared to EVD patients who traveled shorter distance to seek treatment at KGHETC. Also, adjusting for other covariates in the model, older EVD patients (0.21, 95% CI = − 0.36 – 0.77, *p* = 0.57) and EVD patients residing in Kenema District who spent longer time without seeking treatment (− 0.49, 95% CI = − 0.12 – 1.09, *p* = 0.24) had non-significantly reduced LOS before they were discharged alive compared to EVD patients residing in Kenema District who had shorter time without seeking treatment. However, EVD patients who resided in Kenema District (1.21, 95% CI = − 17.67 – 20.09, *p* = 0.89) had non-significantly increased LOS before they were discharged alive following treatment compared to EVD patients who resided outside Kenema District.

### Factors associated with LOS for confirmed EVD patients that died in KGHETC

We determined the factors that are associated with the LOS for 99 EVD patients who died at the KGHETC. There was a non-significant association between occupation levels, EVD patients who resided in Kenema district, delay in seeking EVD treatment, sex and the LOS for confirmed EVD patients admitted at the KGHETC but eventually died during treatment. However, there was a significant association between age, distance traveled to seek treatment by EVD patients and the LOS for confirmed EVD patients admitted at the KGHETC but eventually died during treatment. For EVD patients who died during treatment, adjusting for other covariates in the model, EVD patients with low skills (− 10.33, 95% CI = − 19.57 – - 1.09, *p* = 0.48), children and pupils of junior schools (− 6.17, 95% CI = − 15.23 – 2.87, *p* = 0.80), and EVD patients who resided in Kenema (− 3.34, 95% CI = − 13.93 – 7.25, *p* = 0.52) had non-significantly reduced LOS until death during treatment compared to EVD patients with high skills and those EVD patients who resided outside Kenema district. Additionally, EVD patients residing in Kenema district who spent longer time without seeking treatment (− 0.05, 95% CI = − 0.49 – 0.39, *p* = 0.38) had non-significantly reduced LOS until death during treatment compared to EVD patients residing in Kenema district who spent shorter time without seeking treatment. However, older EVD patients (0.35, 95% CI = 0.11–0.58, *p* = 0.03), and those EVD patients who travelled long distances to seek treatment at KGHETC (− 0.07, 95% CI = − 0.12 – - 0.03, p = < 0.0005) had significantly reduced LOS until death during treatment compared to younger EVD patients and EVD patients who traveled shorter distances to seek treatment at KGHETC. Also, male EVD patients (3.36, 95% CI = − 2.54 – 9.25, p = 0.80) had non-significantly increased LOS until death during treatment compared to female EVD patients (Table 5).

**Table 5:** Determinants of length of stay (in days) during Ebola Virus treatment at Kenema Government Hospital Ebola Treatment Center, Sierra Leone by vital status at discharge during the peak period of the West Africa Ebola outbreak 2013–2016. Results from a multivariate linear regression

Patient characteristics	Regression coefficients of EVD patients who survived	95% CI	Regression coefficients of EVD patients who died	95% CI
Sex: male vs female (ref)	- 1.77	- 8.75 – 5.21	3.36	- 2.54 – 9.25
Occupation: high skilled (ref) workers				
Low skilled workers	- 5.91	- 24.60 – 12.79	- 10.33	- 19.57 – - 1.09
Children and pupils in junior school	- 0.86	- 12.86 – 11.14	- 6.17	- 15.23 – 2.87
Delay (in days) of health seeking behavior for EVD patients residing in Kenema District	- 0.49	- 0.12 – 1.09	- 0.05	- 0.49 – 0.39
Distance (in km) to ETC	- 0.06	- 0.14 – - 0.02	- 0.07	- 0.12 – - 0.03
Referral status: residency outside-vs-in (ref) Kenema District	1.21	- 17.67 – 20.09	- 3.34	- 13.93 – 7.25
Age	0.21	- 0.36 – 0.77	0.35	0.11–0.58

*OR* Odds Ratio, *ETC* Ebola Treatment Center, *EVD* Ebola Virus Disease, *CI* Confidence Interval

## Discussion

We investigated the factors that are associated with the in-facility CFR, odds of dying and the LOS for EVD patients treated at the KGHEC. We described the influence age, occupation type, sex, distance to ETC, and health seeking delay by EVD patients residing in Kenema District had on in-facility CFR; the odds of dying during EVD treatment, and factors associated with the LOS for EVD patients discharged alive as well as for those who died during EVD treatment. Different CFRs were reported for different locations and settings during the 2013–2016 EVD outbreak [1, 8–27]. Worthy of note is that our CFR is different from those of similar studies conducted in Sierra Leone during the said EVD outreak [4, 5, 7, 11, 12, 20, 26–28]. Our high overall CFR compared to the WHO-computed CFR [29] for Sierra Leone for the same outbreak is surprising, as KGH which provided staff for KGHETC during the outbreak was considered to have one of the best facilities in West Africa for EVD case management due to their years of experience in handling Lassa fever cases [30]. Lassa fever and EVD belongs to the same family of enveloped RNA viral pathogens that induce hemorrhagic fever [31] and both of them have similar human-to-human transmission mode and case management strategies. KGHETC suffered from high overall CFR although it was considered at the time as an exemplary facility probably due to poor EVD case management strategies, insufficient and inappropriate logistics as well as from the poor training of KGHETC clinicians during the EVD outbreak period. The KGH Lassa fever program had for years back shifted from patient care to laboratory research work due to under-funding [30, 32]; which made these clinicians ill-prepared to handle EVD cases during the early period of the 2013–2016 EVD outbreak. The lack of the necessary logistics to handle EVD cases may have also overexposed these staffs to the infection which greatly affected their staff strength and led to a cycle of new and more EVD cases and subsequent deaths. Like Fitzpatrick G et al. [4] and Rudolf et al. [33], we also discovered that EVD patients who resided at short distance from the KGHETC alongside early health seeking [33, 34] had a major influence on the in-hospital CFR of EVD patients. The significantly higher CFR among EVD patients who resided in Kenema District compared to EVD patients from other districts who sought EVD treatment at the KGHETC may have been due to the admission of mostly severed EVD patients from within Kenema District who delayed in seeking early EVD treatment. EVD patients from Kenema District had higher delays in health seeking compared to those that came from outside Kenema District to be treated at KGHETC. Health seeking delay could be linked within this context to poor or insufficient health education or the misunderstanding of the health education messages related to the transmission dynamics, mode of infection, control and personal protection against EVD that was passed on at the time of the EVD outbreak. Many residents of Kenema District including staff at the Kenema Government Hospital where the KGHETC was based may have had a false sense of security that previous exposure to Lassa fever either in the community or in the clinical setting makes one immune to EVD. Kenema District is endemic to Lassa fever and records the highest annual incidence of Lassa fever cases in Sierra Leone [35]. This false sense of security may have led to the lack of or poor adherence to both the EVD health messages passed on during the outbreak as well as to that of the EVD infection prevention and control practices [32].

The insufficient or lack of health education on the part of many EVD patients challenged their understanding of the basic principles behind EVD health education messages that were spread at the time of the outbreak, such as infection prevention or early health seeking behaviour. Early treatment as well as the expert knowledge and skills of health practitioners were reported to have been very crucial in controlling and reducing the impact of the Ebola epidemic in Sierra Leone [33, 34]. Stehling-Ariza T and colleagues also attributed the quicker identification of suspected Ebola cases as well as the interruption of Ebola transmission to active case surveillance and health education during the outbreak period [36]. Another possibility for the significantly lower CFR among the EVD patients who resided outside Kenema District compared to those EVD patients who resided within Kenema District is that the more severe EVD cases of those who resided outside Kenema District may have been so weak to not even venture to travel for admission to KGHETC, or that they died before they could reach the KGHETC thereby introducing survival bias [2, 4, 37]. Similarly also, the lower CFR amongst EVD patients who came from outside Kenema District may be attributed to the fact that this subpopulation of EVD patients received decentralized (and hence maybe more attentive) treatment in holding centers [37] en route to KGHETC, thereby providing them additional advantage compared to those EVD patients who resided within Kenema District. It is also possible that EVD cases residing within Kenema District may have presented high Ebola Virus (EV) viral load at the time of admission. Although we did not measure EV viral load upon admission, Theocharopoulus et al. had previously reported that the low EVD viral load of patients from outside the district where the EVD patients was referred from compared to those EVD patients admitted from within the district may be due to their longer admission time, as well as in-transit admission prior to their referral to the distance ETC [26], while some studies have reported high viral load as a significant predictor of EVD mortality [26, 36, 37]. However, like Theocharopoulus et al. [26] we also discovered no significant association between the odds of dying for EVD patients who were residing outside Kenema District and those EVD patients who were residing within Kenema District as well as for those EVD patients who stayed very far from an ETC compared to those who stayed closer to the ETC. Like the establishment of proximal ETCs for EVD patients, the finding that patients who resided within Kenema District had more favourable odds of survival when the sought early treatment once again suggests the importance of information, education and communication (IEC) programs as an essential tool in outbreak management. IEC programs are messaging strategies that have been reported to be effective and crucial in controlling Ebola outbreaks if they are implemented early and involve key stakeholders during an intervention [38]. From our study it becomes apparent that to improve the odds of survival during an EVD treatment, disease prevention and control agencies should prioritize the dissemination of messages about the disease, its treatment availability as well as its control strategies. Additionally, an understanding of the perception and responses to the EVD outbreak by the community should also lie at the core of any EVD outbreak IEC program.

We discovered that EVD patients who came from outside Kenema District where the KGHETC was located spent less time to be cured compared to those who resided within Kenema District. This observation may be due to the admission of EVD patients from within Kenema District that may have been suffering from a more severe course of infection as compared to EVD patients from the other districts. EVD patients with a more severe course of infection and more impaired general condition will have to spend more time to be cured, but may in case of a fatal outcome show a shorter time period until death. Thus, our position is that, although the establishment of a community and proximal ETC is very crucial for reducing community and in-facility nosocomial EVD transmission, yet we believe that seeking early EVD treatment is very important for attaining a positive treatment outcome. We believe that IEC programs alongside with other EVD sensitization and awareness raising strategies that target health benefits of early EVD treatment should be rigorously applied within an EVD outbreak.

Our study is beset with some limitations: (1) Lack of sufficient clinical information that serves as a challenge for conducting an in-depth analysis of any association between CFR, LOS and EVD patient referral status. Unlike other ETCs, like the 34 Military Hospital ETC that started operations in the capital city Freetown in the midway of the 2013–2016 EVD outbreak, the KGHETC was overwhelmed during the study period since they were the first ETC having to start operations in the country. Because of this overstretch at the KGHETC, EVD patients clinical information were collected on a limited basis and was focused on syndromic classifications including but not limited to “admitted in bad state” as well as few striking findings (mutism, blindness, anorexia) that would be relevant in the course of further patient management within the facility. In some patients it is not even clear whether some of the reported clinical symptoms had been present prior to their EVD infection. This limited clinical information in our data rendered an in-depth analysis of the relationship between the spectrum and severity of clinical characteristics of an EVD patient and the evolution of the infection and its treatment outcome challenging. (2) Also, there was no laboratory data (cycle threshold-C_t_) on the EVD viral load of the patients upon their admission. Such data would have provided us another proxy indicator for severity in a given EVD patient upon admission.

## Conclusions

From our findings we observed that health seeking delay, distance travelled to treatment facilities, length of stay and treatment outcomes show complex interdependences. This implies that in any future Ebola outbreak where community EVD transmission is well underway, health authorities should consider targeted health education with high priority given to seeking early EVD treatment, as well as the construction of strategic ETCs as an important component of their response strategy.

## Data Availability

The datasets generated and analyzed during the current study are not publicly available due to patient confidentiality and the sensitive nature of this study. This is an aggregate dataset that is being protected by the Sierra Leone Ethics and Scientific Review Committee in order to protect the identity of the patients whose medical data were analyzed.

## Abbreviations

CFR: Case Fatality Rate
CHD: Cadaver Handling Delay
CI: Confidence Interval
CRF: Case Report Form
DDP: Delay in the Discharge of Patient
DTI: Discharge Testing Interval
ETC: Ebola Treatment Center
EV: Ebola Virus
EVD: Ebola Virus Disease
HSD: Health Seeking Delay for all EVD patients irrespective of District of origin
HSDCP: Health Seeking Delay by EVD Patients who were discharged alive
HSDDP: Health Seeking Delay by EVD patients who Died
HSDKD: Health Seeking Delay for patient residing in Kenema District
HSDNK: Health Seeking Delay for patient residing outside Kenema District
IEC: Information, Education and Communication
IQR: Interquartile Range
KGH: Kenema Government Hospital
KGHETC: Kenema Government Hospital Ebola Treatment Center
LMU: Ludwig-Maximilians-Universität, Munich, Germany
LOS: Length of stay
LOSA: Length of Stay for EVD Patient who Survived Treatment
LOS-CP: Length of stay for EVD patients who were discharged alive
LOS-DP: Length of stay for EVD patients who Died
LSP: Length of Symptomatic Period
OLOS: Overall Length of stay
ORS: Oral Rehydration Salt
RNA: Ribonucleic acid
RRD: Return of EVD test Result Delay
RT-PCR: Real Time Polymerase Chain Reaction
RTR: Return of EVD Test Result
SPCP: Symptomatic period for EVD patients who were discharged alive
SPDP: Symptomatic period for EVD patients who died
